# Glucosamine inhibits IL-1β expression by preserving mitochondrial integrity and disrupting assembly of the NLRP3 inflammasome

**DOI:** 10.1038/s41598-019-42130-z

**Published:** 2019-04-03

**Authors:** Hsiao-Wen Chiu, Lan-Hui Li, Chih-Yu Hsieh, Yerra Koteswara Rao, Fang-Hsin Chen, Ann Chen, Shuk-Man Ka, Kuo-Feng Hua

**Affiliations:** 1 Graduate Institute of Life Science, National Defense Medical Center, Taipei, Taiwan; 2 Department of Laboratory Medicine, Linsen, Chinese Medicine and Kunming Branch, Taipei City Hospital, Taipei, Taiwan; 3 Department of Biotechnology and Animal Science, National Ilan University, Ilan, Taiwan; 4 Department of Medical Imaging and Radiological Sciences, Chang Gung University, Taoyuan, Taiwan; 5 Departments of Pathology, Tri-Service General Hospital, National Defense Medical Center, Taipei, Taiwan; 6 Graduate Institute of Aerospace and Undersea Medicine, Department of Medicine, National Defense Medical Center, Taipei, Taiwan; 7 Department of Medical Research, China Medical University Hospital, China Medical University, Taichung, Taiwan

## Abstract

The NLRP3 inflammasome promotes the pathogenesis of metabolic, neurodegenerative and infectious diseases. Increasing evidences show that the NLRP3 inflammasome is a promising therapeutic target in inflammatory diseases. Glucosamine is widely used as a dietary supplement to promote the health of cartilage tissue and is expected to exert anti-inflammatory activity in joint inflammation, which is a nucleotide-binding oligomerization domain-like receptor containing pyrin domain 3 (NLRP3) inflammasome-associated complication. Here, we investigated whether GlcN inhibits the NLRP3 inflammasome and dissected the underlying molecular mechanisms. We found that GlcN suppressed the NLRP3 inflammasome in mouse and human macrophages. A mechanistic study revealed that GlcN inhibited the expression of NLRP3 and IL-1β precursor by reducing reactive oxygen species generation and NF-κB activation in lipopolysaccharide-activated macrophages. GlcN also suppressed mitochondrial reactive oxygen species generation and mitochondrial integrity loss in NLRP3-activated macrophages. Additionally, GlcN disrupted NLRP3 inflammasome assembly by inhibiting NLRP3 binding to PKR, NEK7 and ASC. Furthermore, oral administration of GlcN reduced peritoneal neutrophils influx and lavage fluids concentrations of IL-1β, IL-6 MCP-1 and TNF-α in uric acid crystal-injected mice. These results indicated that GlcN might be a novel dietary supplement for the amelioration of NLRP3 inflammasome-associated complications.

## Introduction

The nucleotide-binding oligomerization domain-like receptor containing pyrin domain 3 (NLRP3) inflammasome is a protein complex composed of NLRP3, apoptosis-associated speck-like protein containing CARD domain (ASC) and caspase-1 that controls the release of the pro-inflammatory cytokines IL-1β and IL-18^1^. The NLRP3 inflammasome plays important roles in the pathogenesis of metabolic diseases, neurodegenerative diseases and infectious diseases^2^. We previous demonstrated that the NLRP3 inflammasome is involved in the pathogenesis of chronic kidney diseases, including diabetic nephropathy^3^, lupus nephritis^4^, IgA nephropathy^5^ and renal inflammation induced by unilateral ureteral obstruction^6^. A potent and selective small-molecule inhibitor of NLRP3, MCC950, has been developed and shown to attenuate NLRP3-associated syndromes, including experimental autoimmune encephalomyelitis and cryopyrin-associated periodic syndrome, in mice^7^. Additionally, we have identified several compounds that can inhibit the NLRP3 inflammasome *in vitro* and *in vivo*, indicating that the NLRP3 inflammasome is a promising therapeutic target in inflammatory diseases^4,8–10^.

Glucosamine is widely used as a dietary supplement to promote the health of joints and may reduce the pain caused by joint diseases. In addition to its joint-protective effects, glucosamine exerts anti-inflammatory activities and ameliorates inflammatory diseases. For example, glucosamine inhibits inducible nitric oxide synthase expression in lipopolysaccharide (LPS)-activated microglia^11^ and reduces COX-2 expression in human breast cancer cells^12^. Glucosamine exerts *in vivo* anti-inflammatory effects because it ameliorates brain inflammation^11^, inflammatory bowel disease^13^, lung inflammation^14^, atherosclerosis^15^, experimental autoimmune encephalomyelitis^16^ and renal fibrosis^17^. However, the effect of glucosamine on the NLRP3 inflammasome has not been explored yet.

Glucosamine is widely used as a dietary supplement to promote the health of osteoarthritis (OA) and rheumatoid arthritis (RA)^18,19^. OA and RA are the major joint diseases associated with elevated NLRP3 inflammasome^20,21^. Because glucosamine ameliorates NLRP3-associated inflammatory diseases, we hypothesize that the protective effects of glucosamine may be associated with its anti-NLRP3 inflammasome activity. In this report, we investigated whether GlcN inhibits the NLRP3 inflammasome and dissected the underlying molecular mechanisms in macrophages and in a NLRP3-associated mouse disease model.

## Results

### GlcN inhibits the activation of the NLRP3 inflammasome

**Figure 1:**
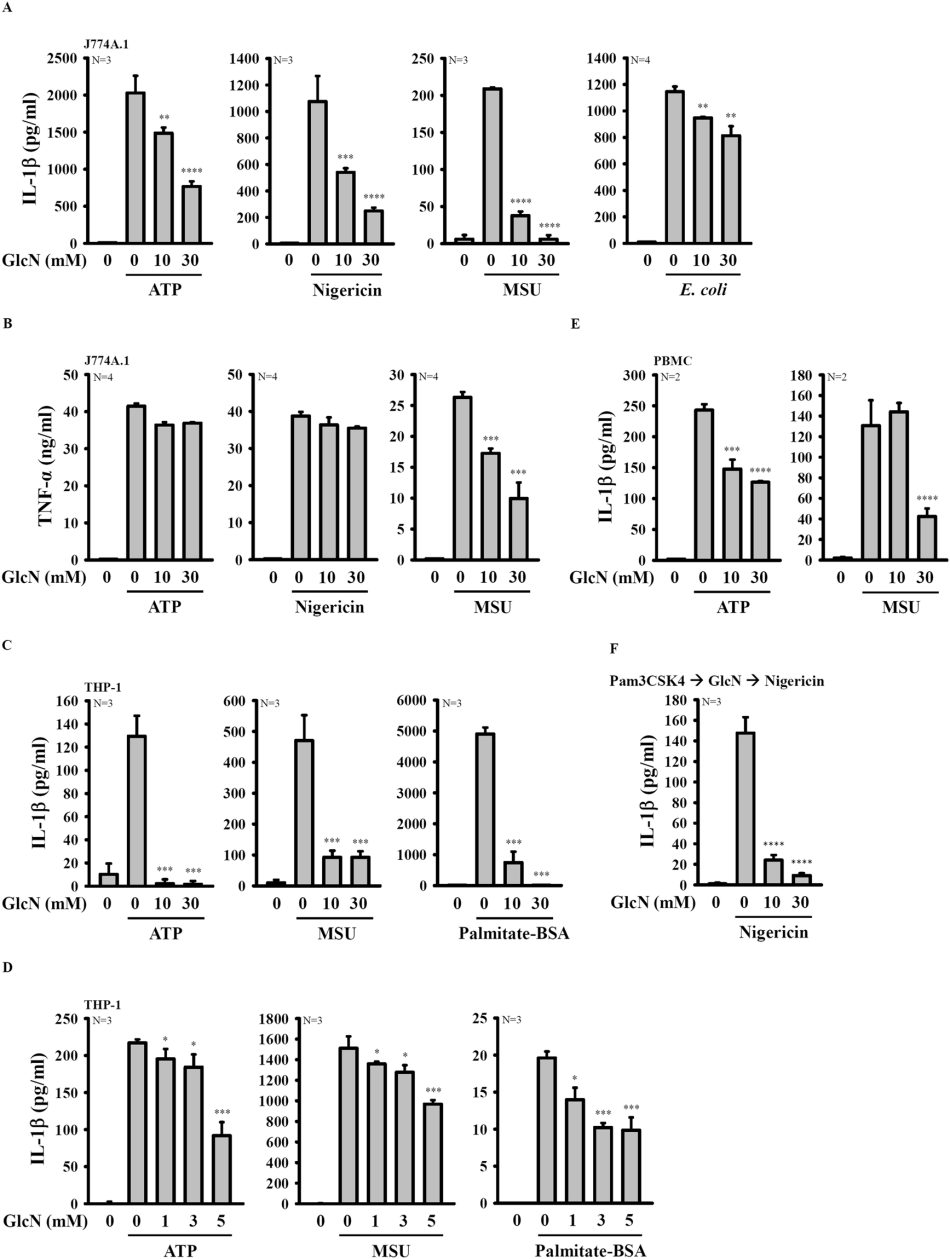
GlcN inhibits activation of the NLRP3 inflammasome. (**A**,**B**) J774A.1 macrophages were incubated for 4 h with LPS (1 µg/ml) followed by incubation for 2 h with GlcN. Cells were then incubated with ATP (5 mM, 0.5 h), nigericin (10 µM, 0.5 h), MSU (100 µg/ml, 24 h) or infected with *E. coli* (30 MOI, 1 h). The IL-1β (**A**) and TNF-α (**B**) expression levels in the supernatants were measured by ELISA. (**C**–**E**) THP-1 macrophages (**C**,**D**) or PBMCs (**E**) were incubated for 4 h with LPS (1 µg/ml) followed by incubation for 2 h with GlcN, followed by incubation with ATP (5 mM, 0.5 h), MSU (100 µg/ml, 24 h) or palmitate-BSA (250 mM, 24 h). The IL-1β expression levels in the supernatants were measured by ELISA. (**F**) J774A.1 macrophages were incubated for 4 h with Pam3CSK4 (1 µg/ml) followed by incubation for 2 h with GlcN. Cells were then incubated with nigericin (10 µM) for 0.5 h. The IL-1β expression levels in the supernatants were measured by ELISA. The ELISA data are expressed as the means ± SD of separate experiments as indicated. *, **, *** and **** indicate a significant difference at the level of *p* < 0.05, *p* < 0.01, *p* < 0.001 and *p* < 0.0001, respectively, compared to NLRP3 activator-treated cells. (One-way ANOVA with Dunnett’s multiple comparisons test).

**Figure 2:**
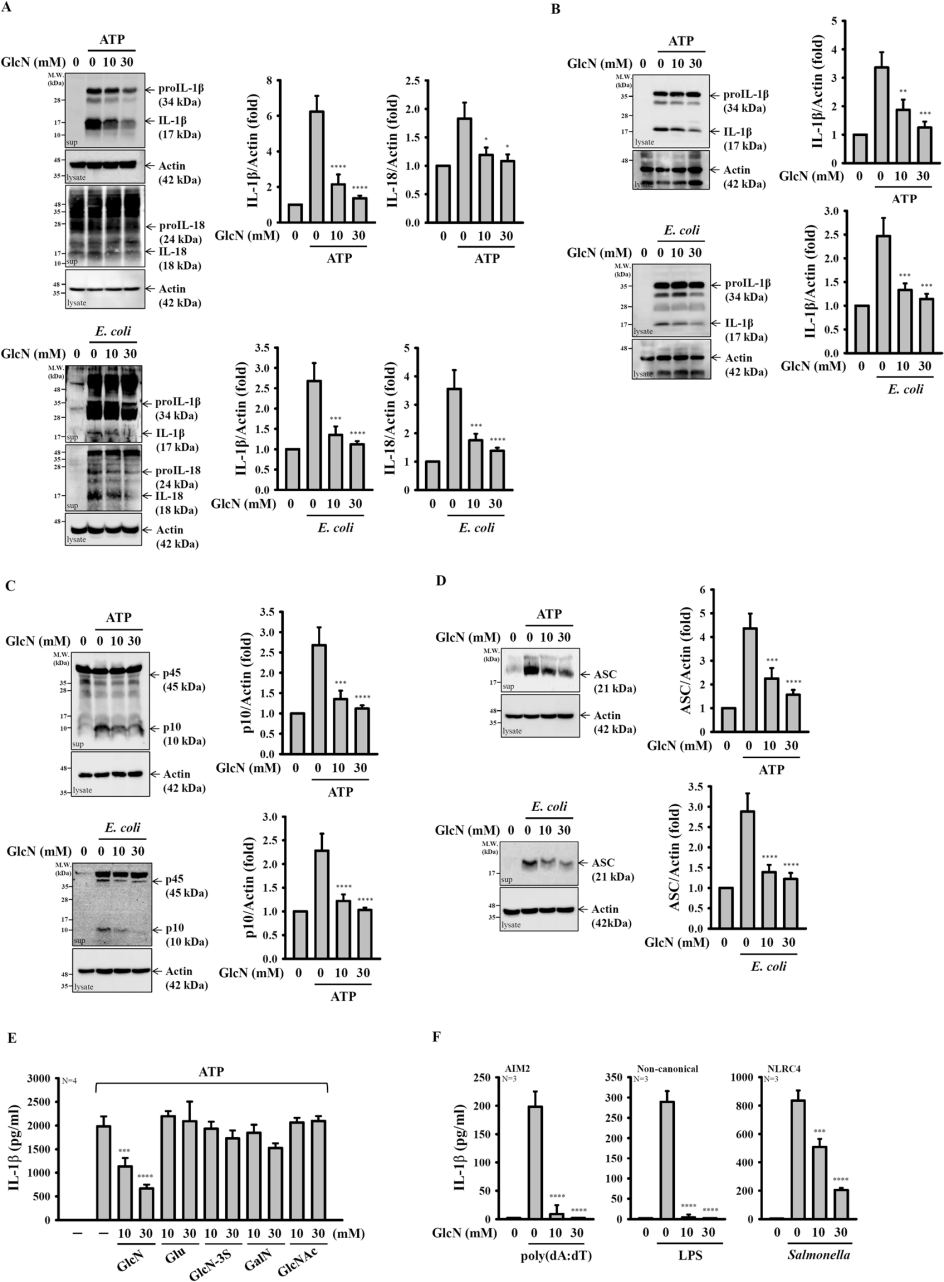
GlcN inhibits caspase-1 activation and the release of IL-1β, IL-18 and ASC. (**A**–**D**) J774A.1 macrophages were incubated for 4 h with LPS (1 µg/ml) followed by incubation for 2 h with GlcN. Cells were then incubated with ATP (5 mM, 0.5 h) or infected with *E. coli* (30 MOI, 1 h). The expression levels of IL-1β and IL-18 (**A**), caspase-1 (**C**), ASC (**D**) in the supernatants and IL-1β in the cell lysates (**B**) were analysed by Western blotting. (**E**) J774A.1 macrophages were incubated for 4 h with LPS (1 µg/ml) followed by incubation for 2 h with GlcN, D-(+)-glucose (Glu), D-glucosamine 3-sulphate (GlcN-3S), D-(+)-galactosamine hydrochloride (GalN) and N-acetyl-D-glucosamine (GlcNAc) for 2 h, followed by incubation with ATP (5 mM) for 0.5 h. The IL-1β expression levels in the supernatants were measured by ELISA. (**F**) J774A.1 macrophages were incubated for 4 h with LPS (1 µg/ml) or PamsCSK4 (1 µg/ml; for non-canonical inflammasome) followed by incubation for 2 h with GlcN, followed by transfection with poly(dA/dT) (2 µg/ml) or LPS (2 µg/ml) for 6 h or by *Salmonella* infection (30 MOI) for 2 h. The IL-1β expression levels in the supernatants were measured by ELISA. The ELISA data are expressed as the mean ± SD of separate experiments as indicated. The Western blotting results are representative of three different experiments and the histogram shows the quantification expressed as the mean ± SD for these three experiments. *, *** and **** indicate a significant difference at the level of *p* < 0.05, *p* < 0.001 and *p* < 0.0001, respectively, compared to NLRP3 activator-treated cells. (One-way ANOVA with Dunnett’s multiple comparisons test). The blots in (**A**–**D**) were cropped from different gels; full-length blots are included in the “Supplementary Information”.

We investigated the inhibition potential of D-(+)-glucosamine hydrochloride (GlcN) toward the NLRP3 inflammasome. First, LPS-primed mouse J774A.1 macrophages were incubated with GlcN for 2 h before stimulation with activators of the NLRP3 inflammasome. We found that GlcN (10 and 30 mM) significantly inhibited IL-1β secretion induced by ATP, nigericin, monosodium urate crystals (MSU) and *E. coli* infection (Fig. 1A). Under the same condition, GlcN did not significantly reduce TNF-α secretion induced by ATP and nigericin, indicating that GlcN specifically inhibited IL-1β secretion (Fig. 1B). GlcN also inhibited NLRP3 inflammasome-derived IL-1β secretion in LPS-primed human THP-1 macrophages (Fig. 1C). GlcN at lower concentration (1, 3 and 5 mM) also inhibited ATP-, MSU- and palmitate-BSA-induced IL-1β secretion in LPS-primed human THP-1 macrophages (Fig. 1D). In addition, GlcN suppressed ATP- and MSU-induced IL-1β secretion in human primary peripheral blood mononuclear cells (PBMCs) (Fig. 1E). To investigate whether the inhibitory effect of GlcN specific to LPS priming only, we investigated the effect of GlcN on the nigericin-induced IL-1β secretion in Pam3CSK4 (toll-like receptor 2 ligand)-primed J774A.1 macrophages. We found that GlcN significantly reduced IL-1β expression levels in the culture medium (Fig. 1F), indicating that the inhibitory effect of GlcN on IL-1β secretion was not specific to LPS priming only. Using Western blotting analysis, we found that GlcN reduced the expression levels of proIL-1β/IL-1β and IL-18 in the supernatants of ATP-activated or *E. coli*-infected J774A.1 macrophages (Fig. 2A). GlcN also inhibited the expression levels of IL-1β in the cell lysates of ATP-activated or *E. coli*-infected J774A.1 macrophages (Fig. 2B). In addition, the inhibitory effect of GlcN on the NLRP3 inflammasome was confirmed by reduced expression levels of active caspase-1 (p10) in the supernatants of ATP-activated or *E. coli*-infected J774A.1 macrophages (Fig. 2C). It has been demonstrated that oligomeric ASC particles are released from macrophages into supernatants upon NLRP3 inflammasome activation and act as an extracellular danger signal that amplifies the inflammatory response^22^. By detection of the expression levels of ASC in the supernatants, we found that ASC particles were released from ATP-activated or *E. coli*-infected J774A.1 macrophages, and this effect was reduced by GlcN (Fig. 2D). Notably, 10 and 30 mM of D-(+)-glucose, D-glucosamine 3-sulphate, D-(+)-galactosamine hydrochloride and N-acetyl-D-glucosamine did not alter IL-1β secretion in ATP-activated macrophages (Fig. 2E). These findings indicated that the NLRP3 inflammasome was inhibited by GlcN, and this effect was not due to the osmotic effect. In addition, to investigate whether GlcN specifically inhibits the NLRP3 inflammasome, the effect of GlcN on the absent in melanoma 2 (AIM2)-, non-canonical- or NLR family CARD domain containing 4 (NLRC4)-inflammasome was tested. The results showed that GlcN not only inhibited NLRP3 inflammasome but also reduced IL-1β secretion in AIM2-, non-canonical- and NLRC4-inflammasome activated J774A.1 macrophages, which are stimulated by poly(dA:dT) and LPS transfection or by *Salmonella* infection, respectively (Fig. 2F).

### GlcN inhibits mitochondrial damage

**Figure 3:**
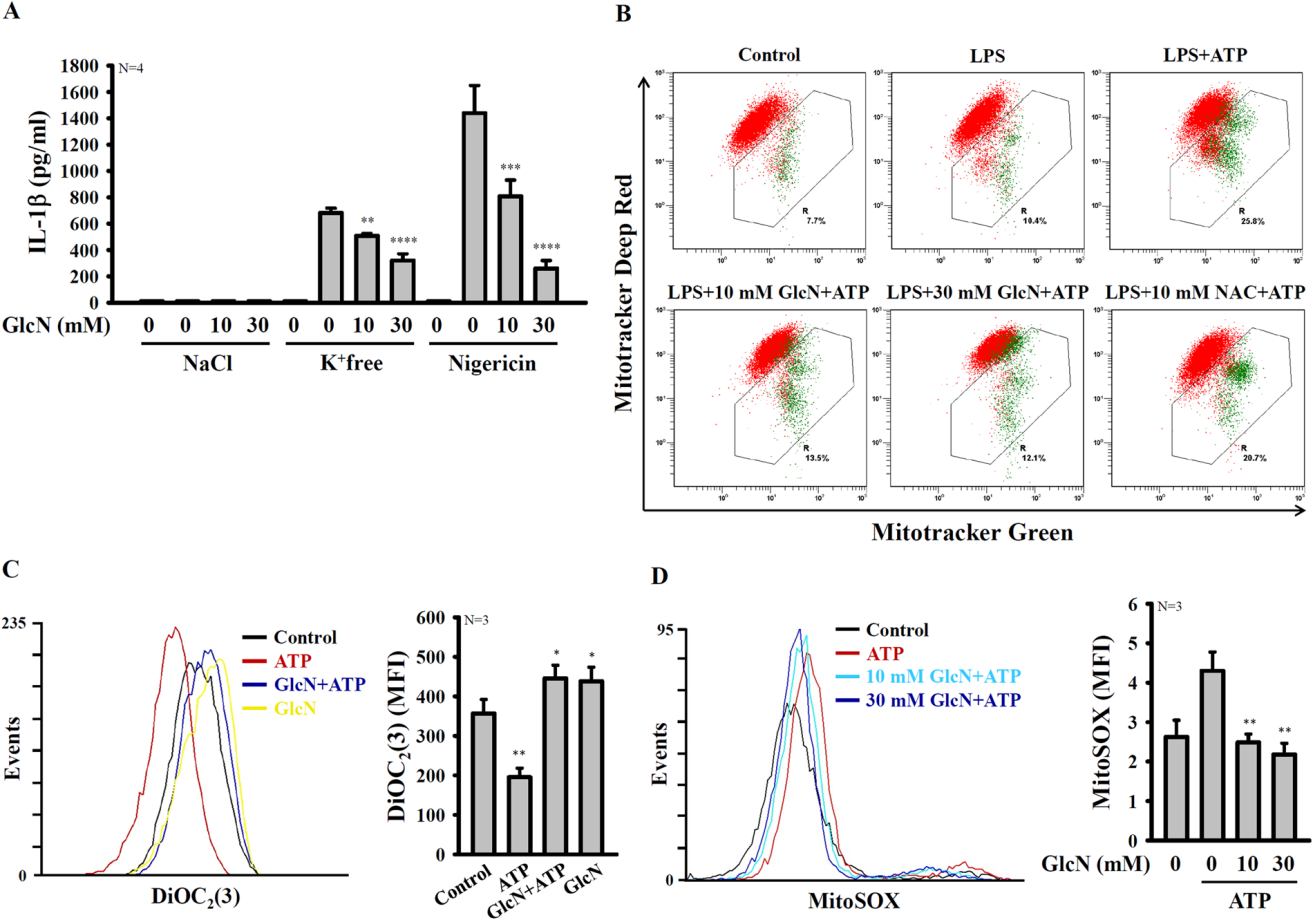
GlcN inhibits K^+^ efflux-induced IL-1β secretion and reduces mitochondrial damage. (**A**) J774A.1 macrophages were incubated for 4 h with LPS (1 µg/ml) followed by incubation for 2 h with GlcN. Cells were then incubated with K^+^-free medium (2 h), NaCl medium (2 h) or nigericin (10 µM, 0.5 h). The IL-1β expression levels in the supernatants were measured by ELISA. (**B**–**D**) J774A.1 macrophages were incubated for 4 h with LPS (1 µg/ml) followed by incubation for 2 h with GlcN or for 0.5 h with NAC. Cells were then incubated with ATP (5 mM) for 0.5 h. Mitochondrial damage was analysed by Mitotracker deep red and Mitotracker green staining (**B**) or DiOC_2_(3) staining (**C**), and mitochondrial ROS production was analysed by MitoSOX red staining (**D**). The data are expressed as the means ± SD of separate experiments as indicated. *, **, *** and **** indicate a significant difference at the level of *p* < 0.05, *p* < 0.01, *p* < 0.001 and *p* < 0.0001, respectively, compared to K^+^-free medium-, nigericin- or ATP-treated cells. (One-way ANOVA with Dunnett’s multiple comparisons test).

K^+^ efflux is the early event of the activation signal of the NLRP3 inflammasome, which activates downstream signalling pathways, leading to caspase-1 activation^23^. To investigate whether GlcN inhibits the NLRP3 inflammasome by targeting the downstream signal of K^+^ efflux, we replaced the culture medium with K^+^ free medium to facilitate K^+^ efflux to promote NLRP3 inflammasome activation. We found that GlcN inhibited IL-1β secretion induced by K^+^ free medium and nigericin, a bacterial toxin that activates the NLRP3 inflammasome through K^+^ efflux, in J774A.1 macrophages (Fig. 3A). These results indicated that GlcN reduced IL-1β secretion by inhibiting the downstream events of K^+^ efflux during NLRP3 inflammasome activation. In addition, mitochondrial damage caused by K^+^ efflux plays important roles in NLRP3 inflammasome activation^24^. We further investigate whether GlcN inhibited NLRP3 inflammasome activation by reducing mitochondrial damage. We found that ATP treatment caused mitochondria damage, as evidenced by reduced Mitotracker deep red staining (Fig. 3B). Treatment with GlcN or antioxidant N-acetyl-L-cysteine (NAC) reversed Mitotracker deep red staining, indicating reduced mitochondrial injury (Fig. 3B). To confirm the effect of GlcN on preserving mitochondrial integrity, DiOC_2_(3), a membrane-potential-sensitive cyanine dye, which penetrates the cytosol of eukaryotic cells and accumulates primarily in mitochondria with active membrane potentials, producing bright, red fluorescence, was used. We found that ATP treatment caused the reduced DiOC_2_(3) staining in LPS-primed macrophages, but this effect was inhibited by GlcN treatment (Fig. 3C). This result indicated that GlcN preserved mitochondrial integrity. Interestingly, GlcN alone increased the DiOC_2_(3) staining compared to the control cells, indicating that GlcN increased mitochondrial function (Fig. 3C). Furthermore, ATP treatment induced mitochondrial ROS production (Fig. 3D), leading to NLRP3 inflammasome assembly^24^. We found that ATP-mediated mitochondrial ROS production was reduced by GlcN treatment in J774 A.1 macrophages (Fig. 3D). These results demonstrated that GlcN inhibited NLRP3 inflammasome activation by reducing mitochondrial integrity loss.

### GlcN inhibits NLRP3 inflammasome assembly

**Figure 4:**
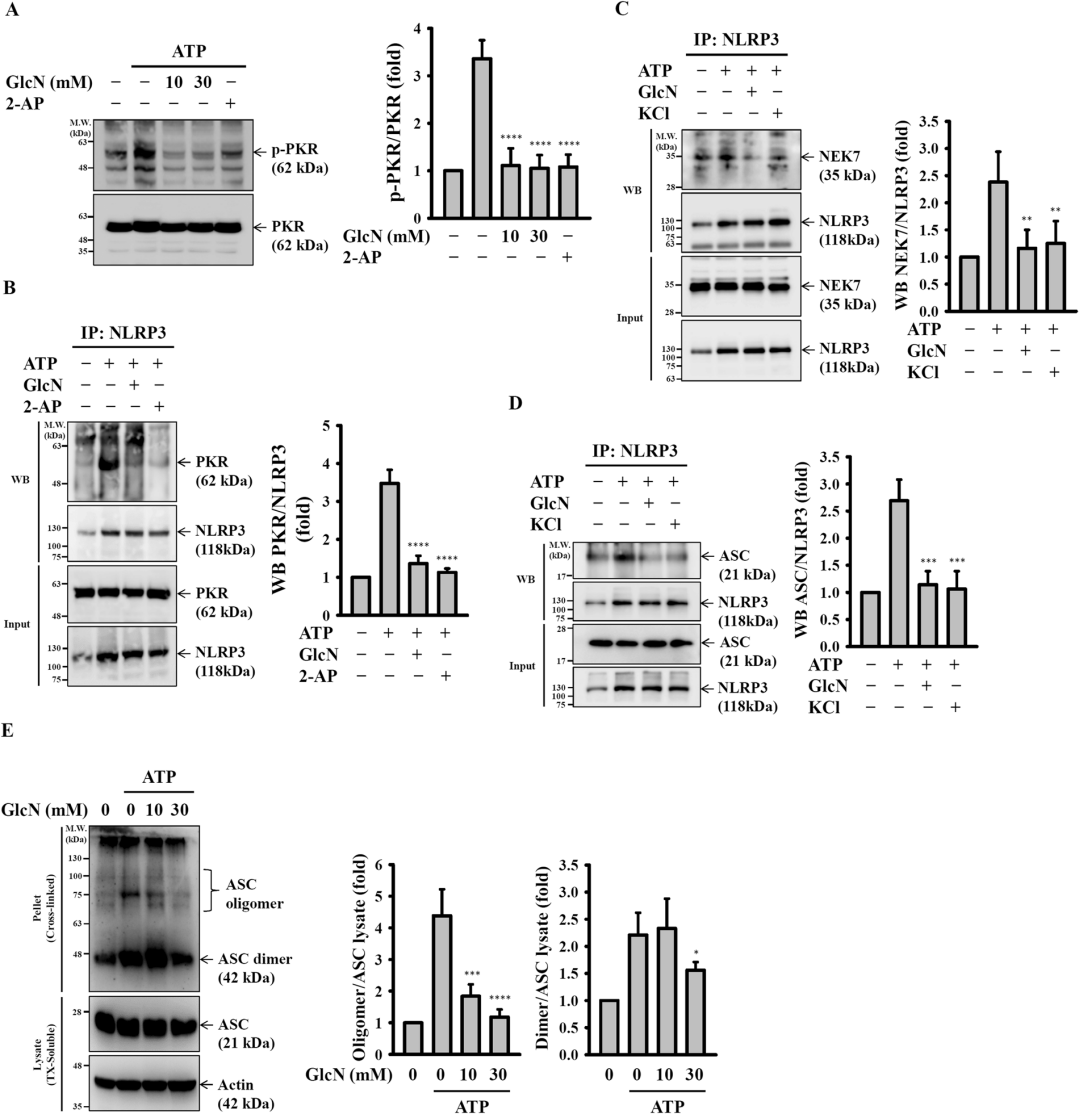
GlcN inhibits NLRP3 inflammasome assembly. (**A**,**B**) J774A.1 macrophages were incubated for 4 h with LPS (1 µg/ml) followed by incubation for 2 h with GlcN or 2-AP (0.5 mM). Cells were then incubated with ATP (5 mM) for 0.5 h. (**A**) The phosphorylation levels of PKR were measured by Western blotting. (**B**) PKR/NLRP3 complex formation was analysed by immunoprecipitation of NLPR3 and Western blotting against PKR. (**C**,**D**) J774A.1 macrophages were incubated for 4 h with LPS (1 µg/ml) followed by incubation for 2 h with GlcN (10 mM) or KCl (50 mM). Cells were then incubated with ATP (5 mM) for 0.5 h. (**C**) NEK7/NLRP3 and (**D**) ASC/NLRP3 complex formation was analysed by immunoprecipitation of NLPR3 and Western blotting against NEK7 and ASC, respectively. (**E**) J774A.1 macrophages were incubated for 4 h with LPS (1 µg/ml) followed by incubation for 2 h with GlcN. Cells were then incubated with ATP (5 mM) for 0.5 h. The cell lysates were crosslinked by disuccinimidyl suberate, and ASC oligomerization was measured by Western blotting. The Western blotting results are representative of three different experiments and the histogram shows the quantification expressed as the mean ± SD for these three experiments. *, **, *** and **** indicate a significant difference at the level of *p* < 0.05, *p* < 0.01, *p* < 0.001 and *p* < 0.0001, respectively, compared to ATP-treated cells. NS: not significant. (One-way ANOVA with Dunnett’s multiple comparisons test). The blots in this figure were cropped from different gels; full-length blots are included in the “Supplementary Information”.

It was demonstrated that activated double-stranded RNA-activated protein kinase (PKR) positively regulates the NLRP3 inflammasome by physically interacting with NLRP3^25^. These results prompted us to investigate the effect of GlcN on PKR activation and the interaction between PKR and NLRP3. We found that the phosphorylation level of PKR was increased by ATP treatment and that this effect was reduced by GlcN or 2-aminopurine (2-AP), a potent inhibitor of PKR (Fig. 4A). Notably, PKR physically interacted with NLRP3 in ATP-stimulated cells, and the interaction was disrupted by GlcN or 2-AP (Fig. 4B). Additionally, NIMA-related kinase 7 (NEK7), an NLRP3-binding protein, is an essential mediator of NLRP3 inflammasome activation downstream of K^+^ efflux^26^. We found that NEK7 binds to NLRP3 upon ATP stimulation and that this effect was suppressed by GlcN and KCl, an inhibitor of K^+^ efflux (Fig. 4C). It was demonstrated that ASC plays key roles in the regulation of the NLRP3 inflammasome via self-association and formation of a complex with NLRP3^27^. We asked whether GlcN affects NLRP3/ASC complex formation. We found that ASC formed a complex with NLRP3 upon ATP stimulation, and this effect was suppressed by GlcN and KCl (Fig. 4D). Furthermore, we found that ATP-induced ASC oligomerization was significantly reduced by GlcN (Fig. 4E). These results indicated that GlcN suppressed NLRP3 inflammasome by inhibiting NLRP3 inflammasome assembly.

### GlcN inhibits the priming signal of the NLRP3 inflammasome by reducing ROS production and NF-κB activation

**Figure 5:**
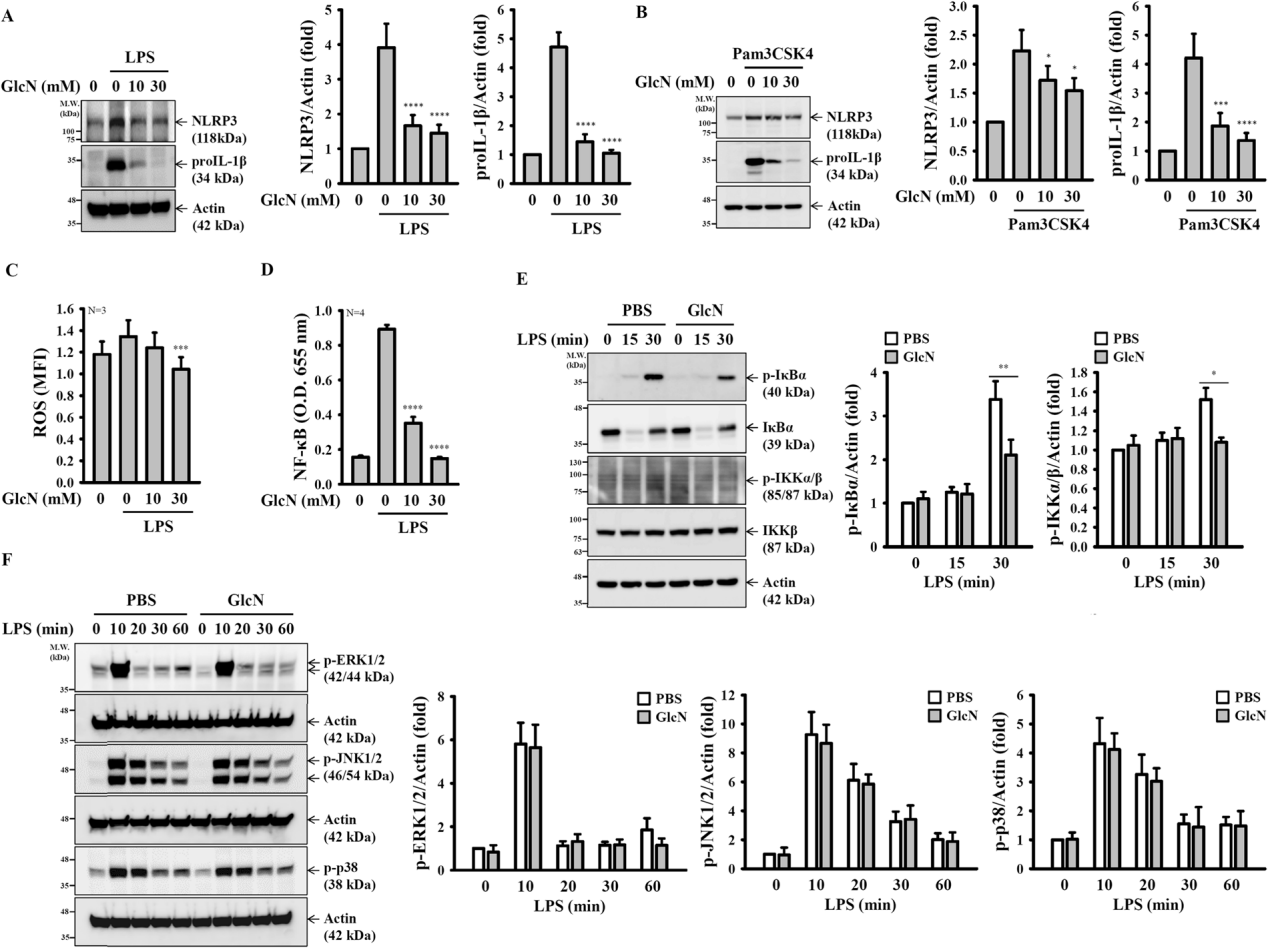
GlcN inhibits the priming signal of the NLRP3 inflammasome. (**A**,**B**) J774A.1 macrophages were incubated with GlcN for 2 h, followed by incubation with LPS (1 µg/ml) (**A**) or Pam3CSK4 (1 µg/ml) (**B**) for 6 h. The expression levels of NLRP3 and proIL-1β were measured by Western blotting. (**C**) J774A.1 macrophages were incubated with GlcN for 2 h, followed by incubation with LPS (1 µg/ml) for 0.5 h. The expression levels of ROS were measured by CM-H_2_DCFDA staining N = 3. (**D**) NF-κB reporter cells were incubated with GlcN for 2 h, followed by incubation with LPS (1 µg/ml) for 24 h. The NF-κB transcriptional activity was assayed by the QUANTI-Blue method N = 4. (**E**) J774A.1 macrophages were incubated with GlcN (10 mM) for 2 h, followed by incubation with LPS (1 µg/ml) for 0–30 min. The expression levels of p-IκBα, IκBα, p-IKKα/β and IKKβ were measured by Western blotting. (**F**) J774A.1 macrophages were incubated with GlcN (10 mM) or PBS for 2 h, followed by incubation with LPS (1 µg/ml) for 0–60 min. The phosphorylation levels of MAPKs were measured by Western blotting. The Western blotting results are representative of three different experiments and the histogram shows the quantification expressed as the mean ± SD for these three experiments. *, **, *** and **** indicate a significant difference at the level of *p* < 0.05, *p* < 0.01, *p* < 0.001 and *p* < 0.0001, respectively, compared to LPS-treated cells (**A**–**D**) or as indicated (**E**,**F**). (ANOVA with Dunn’s multiple comparisons test in **A**–**D**; or two-tailed t test in **E** and **F**). The blots in (**A**), (**B**), (**E**) and (**F**) were cropped from different gels; full-length blots are included in the “Supplementary Information”.

In addition to the activation signal, full activation of the NLRP3 inflammasome required the priming signal from pathogen-associated molecular patterns, such as LPS, which transcriptional control the expression of NLRP3 and proIL-1β^28^. To investigate whether the priming signal of the NLRP3 inflammasome was affected by GlcN, LPS- and Pam3CSK4-mediated NLRP3 and proIL-1β expression in GlcN-treated J774A.1 macrophages were measured by Western blotting. We found that the NLRP3 and proIL-1β expression levels in both LPS- and Pam3CSK4-activated macrophages were significantly inhibited by GlcN (Fig. 5A,B), indicating that GlcN inhibited the priming signal of the NLRP3 inflammasome. ROS has been demonstrated to act as a critical event upstream of the LPS-mediated priming signal of the NLRP3 inflammasome^29^. We found that GlcN significantly inhibited ROS production in LPS-activated macrophages (Fig. 5C). In addition, NF-κB activation is also required for LPS-mediated NLRP3 and proIL-1β expression^30^. In this study, we found that LPS increased the transcriptional activity of NF-κB, and this effect was reduced by GlcN (Fig. 5D). To provide additional evidences for GlcN-mediated NF-κB inhibition, we investigated the effect of GlcN on LPS-induced phosphorylation of IκBα and IKKα/β in J774A.1 macrophages. We found that the phosphorylation levels of IκBα and IKKα/β were reduced by GlcN (Fig. 5E). However, the results showed that GlcN had no effect on the phosphorylation levels of ERK1/2, JNK1/2 and p38 (Fig. 5F), which are important signalling molecules for NLRP3 and proIL-1β expression in LPS-activated macrophages^31^.

### GlcN inhibits the NLRP3 inflammasome and inflammation in MSU-injected mice

**Figure 6:**
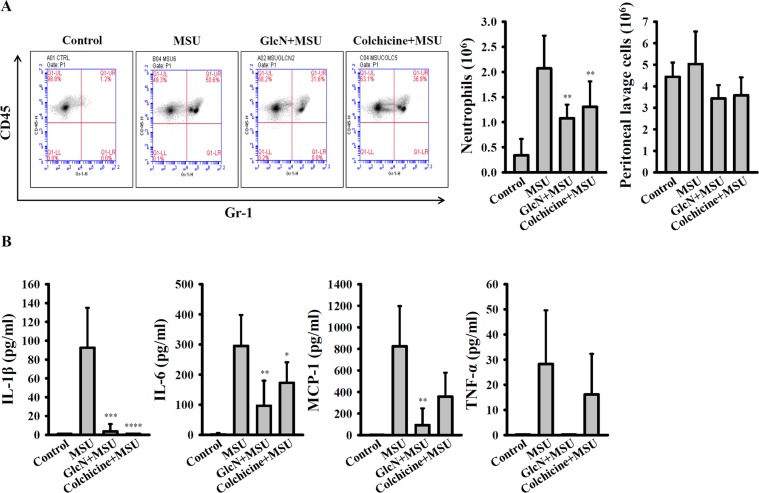
GlcN inhibits the NLRP3 inflammasome in the mouse model of uric acid crystal-mediated peritonitis. (**A**) Peritoneal recruitment of neutrophils and total peritoneal lavage cells were measured by flow cytometry. (**B**) The peritoneal levels of IL-1β, IL-6, MCP-1 and TNF-α were measured by ELISA. Control group: N = 3; MSU group: N = 10; GlcN + MSU group: N = 10; Colchicine + MSU group: N = 8. *, **, *** and **** indicate a significant difference at the level of *p* < 0.05, *p* < 0.01, *p* < 0.001 and *p* < 0.0001, respectively, compared to MSU group. (One-way ANOVA with Dunnett’s multiple comparisons test).

To investigate whether GlcN exhibits anti-NLRP3 inflammasome activity *in vivo*, an NLRP3-associated inflammatory disease mouse model of MSU-mediated peritonitis was used^32^. As can be seen in Fig. 6A, MSU intraperitoneal injection (i.p.) caused significant peritoneal neutrophils influx; notably, oral administration of 250 mg/kg GlcN (three times daily before MSU injection) or i.p. of colchicine (once before MSU injection) significantly reduced peritoneal neutrophils influx. However, the total numbers of peritoneal lavage cells were no significant difference (Fig. 6A). MSU injection also increased the levels of IL-1β, IL-6, TNF-α and MCP-1 in the peritoneal fluid, and these effects were reduced by GlcN and colchicines (Fig. 6B). These findings indicated that GlcN inhibits the NLRP3 inflammasome and inflammation *in vivo*.

## Discussion

The NLRP3 inflammasome is an important focus of biomedical research, and the number of NLRP3-related studies is rapidly growing. The NLRP3 inflammasome plays crucial roles in the pathogenesis of metabolic, autoimmune, neurodegenerative diseases as well as in host defence against invading pathogens^2^. Over the past decade, research efforts have focused on understanding the regulation of the NLRP3 inflammasome; currently, it has become a promising molecular target in the fight against inflammatory diseases^33^. We identified several small-molecule inhibitors of the NLRP3 inflammasome from natural products that ameliorate NLRP3-associated disease *in vitro* and *in vivo*. Citral and Epigallocatechin-3-gallate from *Litsea cubeba* and green tea, respectively, prevent lupus nephritis in mice^4,34^. Resveratrol, osthole and antroquinonol from the skin of red grapes, *Cnidiummonnieri* (L.) Cusson seeds and the *Antrodia camphorata* mycelium, respectively, mitigate IgA nephropathy in mice^9,10,35^. A cinnamaldehyde derivative from the essential oil of the leaves of *Cinnamomum osmophloeum* kaneh ameliorates LPS-induced renal inflammation in mice^36^. In addition, two small-molecule inhibitors of the NLRP3 inflammasome, MCC950 and β-hydroxybutyrate, were reported in *Nature Medicine* 2015 and showed promise in the clinic^7,37^. These results indicated that small molecules have the potential to be developed into NLRP3-targeting therapeutics against various NLRP3 inflammasome-associated diseases.

We demonstrated that GlcN inhibited the NLRP3 inflammasome, machinery that plays important roles in the pathogenesis of diabetes and atherosclerosis^38,39^. These results suggested that GlcN may ameliorate diabetes and atherosclerosis by reducing NLRP3 inflammasome activation. However, there are little data regarding the side effects of GlcN supplementation on metabolic or vascular systems. It was reported that GlcN promotes endoplasmic reticulum stress in retinal Müller cells and leads to the resistance to insulin action, suggesting that GlcN potentially exacerbates diabetes-induced hyperglycaemia^40^. Another study also indicated that GlcN enhanced insulin resistance in mice fed a normal diet but ameliorated obesity and insulin resistance in mice fed a high-fat diet^41^. In addition, GlcN supplementation promotes endoplasmic reticulum stress, hepatic steatosis and accelerated atherosclerosis in apolipoprotein E-deficient mice, and these effects are independent of detectable changes in the levels of glucose, insulin and lipid in blood^42^. However, another report indicated that long-term GlcN supplementation was safe and no increase in atherosclerosis occurred, although increased hyperlipidaemia was observed^43^. These controversial findings suggest that the integrative function of GlcN may depend on the physiological or nutrient status.

Our current findings demonstrated that GlcN inhibited the priming signal of the NLRP3 inflammasome partially through reducing LPS-induced ROS production in macrophages. The anti-oxidative effect of GlcN was observed in a mouse model of cigarette smoke-induced lung inflammation^14^. Our previous study indicated that the LPS-mediated priming signal of the NLRP3 inflammasome was partially through MAPK^31^; however, LPS-induced MAPK activation was not inhibited by GlcN. In the activation signal of the NLRP3 inflammasome, K^+^ efflux plays important roles by triggering downstream signalling, leading to activation of the NLRP3 inflammasome^23,24^. Although we had no solid evidence showing the effect of GlcN on the P_2_X_7_ receptor, a nonselective cationic channel abundantly expressed in macrophages that allows the efflux of K^+^ ^44^, we demonstrated that GlcN inhibited downstream signalling of K^+^ efflux, thereby inhibiting IL-1β secretion (Fig. 3A). Mitochondrial damage is one of the downstream signalling events of K^+^ efflux, which is characterized by mitochondrial ROS production and the loss of the mitochondrial inner transmembrane potential^24^. Mitochondrial ROS promotes the translocation of chloride intracellular channels to the plasma membrane for the induction of chloride efflux to promote NLRP3 inflammasome assembly, caspase-1 activation and IL-1β secretion^45^. We found that GlcN inhibited mitochondrial ROS production (Fig. 3D) and NLRP3 inflammasome assembly (Fig. 4); however, the effect of GlcN on chloride efflux needs further investigation. It should be noted that several events associated with the NLRP3 inflammasome activation were inhibited by GlcN. It is important to know whether GlcN had multiple targets or it just inhibited one major molecule and others are just a consequence. According to our previous study^31^, ROS is the key molecule controlling both priming and activating signals of the NLRP3 inflammasome. Our current findings demonstrated that GlcN inhibited the NLRP3 inflammasome by affecting both priming and activating events. We speculated that ROS may be the major molecule that inhibited by GlcN and other events such as reduced NF-κB activation and inflammasome assembly might be the consequence of ROS inhibition. However, more studies are needed to support the hypothesis.

Autophagy is cellular machinery that protects cells from damage. Recent reports have indicated that autophagy inhibits the NLRP3 inflammasome by reducing mitochondrial integrity loss and targeting inflammasome components for lysosomal degradation^46^. A previous study indicated that GlcN induced autophagy in chondrocytes *in vitro* and in articular cartilage *in vivo*^47^. Thus, we speculate that GlcN-mediated inhibition of the NLRP3 inflammasome may partially occur through autophagy induction in macrophages. Notably, LPS-induced TNF-α production was not significantly inhibited by GlcN in ATP- or nigericin-treated cells, but inhibited significantly in MSU-treated cells (Fig. 1B). The reduced TNF-α expression level in MSU-treated cells was due to the experimental condition. In the ATP- or nigericin-treated cells, cells exposed to LPS for total 6.5 h and exposed to GlcN only for the last 2.5 h. However, in the MSU-treated cells, cells exposed to LPS for total 30 h and exposed to GlcN for the last 26 h. We suggested that the reduced TNF-α in MSU-treated cells may result from (1) GlcN inhibited the LPS-mediated signal in the last 26 h; and (2) GlcN-induced autophagy that reduces TNF-α production.

**Figure 7:**
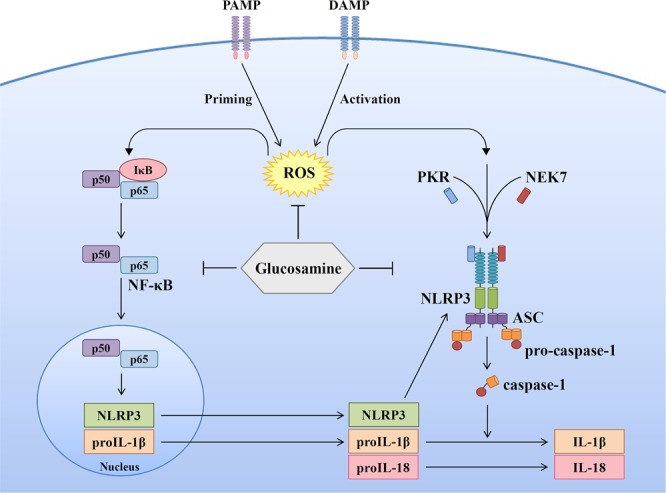
Overview of the putative mechanisms by which GlcN attenuated the NLRP3 inflammasome.

Oral administration of GlcN reduced the peritoneal neutrophils influx and cytokine expression in the peritoneal fluid; however, the total numbers of peritoneal lavage cells were not significantly reduced by GlcN treatment (Fig. 6). The peritoneal lavage cells include not only neutrophils, but also epithelial cells, lymphocytes, plasma cells, monocytes, macrophages and mesothelial cells etc. In our current study we found that while peritoneal recruitment of neutrophils was reduced by GlcN in MSU-injected mice, total peritoneal lavage cells seem to be similar. This result indicated that GlcN specifically reduced the neutrophils influx, but not overall reduced the total number of peritoneal lavage cells. In the *in vivo* study, both GlcN and colchicine reduced the peritoneal neutrophils influx and cytokine expression in the peritoneal fluid, and GlcN was found to be more potent than colchicine in cytokine inhibition (Fig. 6). One of the possibilities is that mice orally administered with GlcN once daily for three days, but i.p. injection with colchicine only once before MSU injection. In previous studies, plasma concentrations of GlcN in rats or human treated with GlcN were increased to dozens to hundreds μM^48,49^. Although we demonstrated that GlcN at 1 mM can inhibit NLRP3 inflammasome in human THP-1 macrophages (Fig. 1C), the GlcN concentration is still higher than the physiological condition. However, many studies demonstrated that GlcN exerted benefit effects *in vivo*^11,13–17^. We speculated that daily low dose GlcN exposure *in vivo* may inhibit the NLRP3 inflammasome. In addition, our current finding provided a possibility to use GlcN as a core structure to make derivatives with enhanced activity^50^. Additionally, as exciting finding is that GlcN extends the life span of nematodes and mice by activating AMP-activated protein kinase^51^. However, it should be noted that GlcN may induce renal toxicity in mice^52^. Therefore, although we provide evidence that GlcN exerts anti-NLRP3 inflammasome activity *in vitro* and *in vivo*, more studies should be performed before using GlcN as a therapeutic agent for NLRP3-associated diseases. Taken together, we found that GlcN might be a novel dietary supplement for the amelioration of NLRP3 inflammasome-associated complications by suppressing both priming and activation signals of the NLRP3 inflammasome (Fig. 7).

## Materials and Methods

### Materials

D-(+)-Glucosamine hydrochloride (G1514), D-(+)-glucose (G8644-100ML), D-glucosamine 3-sulphate (11631), D-(+)-galactosamine hydrochloride (G1639), N-acetyl-D-glucosamine (A3286), LPS (*Escherichia coli* O111:B4, L2630), N-acetyl-L-cysteine (A9165), 2-aminopurine (A3509), palmitic acid (P0500) and Histopaque-1077 (10771) were purchased from Sigma-Aldrich (St. Louis, MO). ATP (tlrl-atp), nigericin (tlrl-nig), monosodium urate (tlrl-msu), NF-κB-inducible reporter plasmid (pnifty2-seap) and QUANTI-Blue medium (rep-qb2) were purchased from InvivoGen (San Diego, CA). Antibodies against β-actin (sc-47778) and ASC (sc-25514-R) were purchased from Santa Cruz Biotechnology (Santa Cruz, CA). Antibodies against caspase-1 (AG-20B-0044) and NLRP3 (AG-20B-0014) were purchased from Adipogen Life Science (San Diego, CA). Antibodies against phospho-MAPK (#9910) were purchased from Cell Signaling Technology (Beverly, MA). Antibodies against IL-18 (ab71495), phospho-PKR (ab32036) and PKR (ab184257) were purchased from Abcam (Cambridge, UK). The antibody against IL-1β (AB-401-NA) was purchased from R&D systems (Minneapolis, MN). ELISA kits were purchased from Affymetrix eBioscience (San Diego, CA). Mitotracker deep red (M22426), Mitotracker green (M7514), MitoSOX red (M36008), disuccinimidyl suberate (21655), CM-H_2_DCFDA (C6827) and DiOC_2_(3) (M34150) were purchased from Thermo Fisher Scientific (Waltham, MA). Phorbol 12-myristate 13-acetate (524400) was purchased from Merck Millipore (Bedford, MA). Bio-Rad Protein Assay Dye Reagent Concentrate (5000006) was purchased from Bio-Rad Laboratories, Inc (Hercules, CA). GeneJammer transfection reagent was purchased from Agilent Technologies (Santa Clara, CA).

### Cell culture

The mouse macrophage cell line J774A.1 and human monocytic leukaemia cell line THP-1 were purchased from the American Type Culture Collection (Rockville, MD). We used whole blood from healthy volunteers recruited at the Tri-Service General Hospital in Taipei, Taiwan, after their approval according to the Institutional Review Board of the Tri-Service General Hospital, National Defense Medical Center and the patients’ informed consent (TSGH-IRB-2-106-05-190 and TSGH-IRB-2-106-05-009). Human primary PBMCs were separated from whole blood from healthy volunteers by density gradient centrifugation using Histopaque-1077 within 24 h after blood was collected, and all experimental protocols were performed in accordance with the guidelines and regulations provided and accepted by the Institutional Review Board of the Tri-Service General Hospital, National Defense Medical Center. In the glucose-treated cells of Fig. 2E, the cells were propagated in glucose free RPMI-1640 medium (catalog number: 11879020, Thermo Fisher Scientific, Waltham, MA) supplemented with 10- or 30-mM D-(+)-glucose (catalog number: G8644-100ML, Sigma, St. Louis, MO). In other studies, the cells were propagated in RPMI-1640 Medium, HEPES (catalog number: 22400089, Thermo Fisher Scientific, Waltham, MA), which contains 2 g/L glucose. All cells were cultured in medium with 10% heat-inactivated foetal bovine serum at 37 °C in a 5% CO_2_ incubator. THP-1 cells were cultured for 48 h in RPMI-1640 medium supplemented with 100 nM phorbol 12-myristate 13-acetate to induce monocyte-to-macrophage differentiation.

### Activation of the inflammasomes

For NLRP3 inflammasome, cells were primed for 4 h with 1 µg/ml LPS followed by treatment for 2 h with GlcN. Cells were then treated with 5 mM ATP or 10 µM nigericin for 0.5 h, or 100 µg/ml MSU and 250 mM palmitate-BSA for 24 h. For AIM2 and non-canonical inflammasome activation, cells were primed for 4 h with 1 µg/ml LPS or 1 µg/ml Pam3CSK4 (for non-canonical inflammasome) followed by treatment for 2 h with GlcN. Cells were then transfection with 2 µg/ml poly(dA/dT) or 2 µg/ml LPS for 6 h, respectively, by GeneJammer transfection reagent. The IL-1β levels in the supernatants were analysed by ELISA, and the levels of proIL-1β/IL-1β, IL-18, caspase-1 (p10 and p45) and ASC in the cell lysates or supernatants were analysed by Western blotting.

### Western blotting analysis of protein expression in the culture medium and lysates

For detection of protein expression in the culture medium, a mixture of 300 μl culture medium, 300 μl methanol, and 125 μl chloroform was prepared. After vortexing, 300 μl double-distilled water was added to the mixture and incubated for 10 min on ice before centrifugation for 10 min at 13,000 rpm at 4 °C. The supernatant was removed, and 500 μl methanol was added; after vortexing, the mixture was centrifuged for 10 min at 13,000 rpm at 4 °C, and then, the supernatant was removed again. The pellet was dried at 55 °C and dissolved in Western blotting loading buffer, followed by incubation in boiling water for 30 min. The samples were analysed by Western blotting. For detection of protein expression in the lysates, ice-cold phosphate-buffered saline (PBS)-washed cells were lysed with 100 μl ice-cold lysis buffer (20 mM Tris-HCl (pH 7.5), 150 mM NaCl, 1 mM EDTA, 1 mM EGTA, 1% NP-40, 1% sodium deoxycholate, 2.5 mM sodium pyrophosphate, 1 mM β-glycerolphosphate, 1 mM Na_3_VO_4_, 1 μg/ml leupeptin, and 1 mM PMSF) on ice for 10 min. Insoluble material was removed by centrifuging at 4 °C for 15 min at 12000 *g*. The protein concentrations were determined using Bio-Rad protein assay dye. 50 μg proteins form each sample was analysed by Western blotting. The bands intensities from each blot were quantified by densitometric analysis using ImageJ software. The densitometry fold change of each group was calculated by comparing the results with the control group. The band density is normalized to actin or indicated protein before fold change is calculated.

### Bacterial strains and infection

*Escherichia coli* (*E. coli*) (ATCC 25922) and *Salmonella* (ATCC 14028) were purchased from the American Type Culture Collection (Rockville, MD). *E. coli* and *Salmonella* were cultured on Eosin Methylene Blue agar or Salmonella Shigella agar, respectively (Creative, TW) at 37 °C in a 5% CO_2_ incubator, and the number of viable bacteria was determined by a colorimeter (M6+ Colorimeter, Metertech). LPS-primed J774A.1 macrophages were treated with GlcN (10, 30 mM) for 2 h and infected 1 h with *E. coli* at a multiplicity of infection (MOI) of 30 or infected 2 h with *Salmonella* at MOI of 20.

### Detection of mitochondrial damage and ROS production

Plate 2 × 10^5^ cells per well in 0.5 ml of culture medium and were grown overnight at 37 °C in a 5% CO_2_ incubator. The cells were primed with 1 μg/ml LPS for 4 h, followed by GlcN (10, 30 mM) treatment for 2 h. The cells were incubated with Mitotracker deep red (25 nM) plus Mitotracker (25 nM) or MitoSOX red (5 nM) for 15 min and, after stimulation, with ATP (5 mM) for an additional 30 min. For DiOC_2_(3) staining, cells were incubated with DiOC_2_(3) (50 nM) in PBS for 15 min after ATP treatment. The fluorescence signal was analysed by flow cytometry (Cytomics FC500 Flow Cytometry CXP, Beckman Coulter Life Sciences).

### ASC oligomerization assay

J774A.1 macrophages were primed for 4 h with 1 µg/ml LPS followed by incubation for 2 h with GlcN. Cells were incubated with 5 mM ATP for additional 0.5 h, and then lysed with TBS buffer (50 mM Tris-HCl (pH 7.4) and 150 mM NaCl) containing 0.5% Triton X-100, EDTA-free protease inhibitors and phosphatase inhibitors. The lysates were centrifuged at 6,000 g for 15 min at 4 °C, and the pellets and supernatants were used as the Triton-insoluble fractions and Triton-soluble fractions, respectively. For the detection of ASC oligomerization, the Triton-insoluble pellets were washed twice with TBS buffer and then were re-suspended in 300 μl TBS buffer. The re-suspended pellets were crosslinking for 30 min with 2 mM disuccinimidyl suberate at 37 °C and then were centrifuged for 15 min at 6,000 g. The pellets were dissolved in SDS sample buffer and processed for SDS-PAGE. The ASC oligomerization were then analysed by Western blotting.

### NF-κB transcriptional assay

J774A.1 macrophages were stably transfected with the pNiFty2-SEAP plasmid, which is a NF-κB-inducible reporter plasmid used for NF-κB transcriptional assay. Plate 2 × 10^5^ cells per well in 0.5 ml of culture medium and were grown overnight at 37 °C in a 5% CO_2_ incubator. The cells were incubated with GlcN (10, 30 mM) for 2 h and then treated with 1 μg/ml LPS for 24 h. The NF-κB transcriptional activity was analysed using QUANTI-Blue medium as described previously^53^.

### Palmitate-BSA complex preparation

Palmitic acid was dissolved in 95% ethanol at 60 °C at a concentration of 300 mM and then was mixed with 5% fatty acid-free bovine serum albumin in PBS to a final concentration of 15 mM.

### Mouse model of uric acid crystal-mediated peritonitis

Seven-to-nine-week-old male C57BL/6JNal mice were purchased from the National Laboratory Animal Center (Taipei, Taiwan) and housed in a room under controlled temperature (23 ± 3 °C) and relative humidity (50 ± 5%). Animal experiments were performed with the approval of the Institutional Animal Care and Use Committee of the National Ilan University (approval number: No. 106-13) according to the NIH Guide for the Care and Use of Laboratory Animals. Mice were randomized into four groups: Normal control (oral administration of 200 μl sterile ddH_2_O (vehicle) at 0, 24 and 48 h; one i.p. injection with 0.5 ml sterile PBS at 50 h; n = 3); Disease group (oral administration of 200 μl sterile ddH_2_O (vehicle) at 0, 24 and 48 h; one i.p. injection with 0.5 ml sterile MSU (3 mg in PBS) at 50 h; n = 10); GlcN treatment group (oral administration of 200 μl sterile GlcN (250 mg/kg) at 0, 24 and 48 h; one i.p. injection with 0.5 ml sterile MSU (3 mg in PBS) at 50 h; n = 10); Colchicine treatment group (one i.p. injection with 0.5 ml sterile colchicine (1 mg/kg) at 48 h, one i.p. injection with 0.5 ml sterile MSU (3 mg in PBS) at 50 h, n = 8). Mice were euthanized at 54 h, and the peritoneum was lavaged with 3 ml of ice-cold sterile PBS. The peritoneal lavage fluid and cells were collected for further analysis.

### Statistical analysis

GraphPad Prism 7.0 software was used for data analysis. Data are shown as mean ± SD. Statistical significance was determined by t tests (two-tailed) for two groups or ANOVA (with Dunnett’s multiple comparisons test) for three or more groups. *P* values less than 0.05 were considered to be statistically significant.

## Supplementary information

Supplementary Info
