# Supplementary material for: Glucosamine inhibits IL-1β expression by preserving mitochondrial integrity and disrupting assembly of the NLRP3 inflammasome
Source: Sci Rep. 2019 Apr 3;9:5603. doi: 10.1038/s41598-019-42130-z (PMC6447579; doi:10.1038/s41598-019-42130-z)

**Supplementary Information for**

**Glucosamine inhibits IL-1β expression by preserving mitochondrial integrity and disrupting assembly of the NLRP3 inflammasome**

Hsiao-Wen Chiu^1^, Lan-Hui Li^2^, Chih-Yu Hsieh^3^, Yerra Koteswara Rao^3^, Fang-Hsin Chen^4^, Ann Chen^5^, Shuk-Man Ka^6,*^, Kuo-Feng Hua^3,5,7,*^

^1^Graduate Institute of Life Science, National Defense Medical Center, Taipei, Taiwan

^2^Department of Laboratory Medicine, Linsen, Chinese Medicine and Kunming Branch, Taipei City Hospital, Taipei, Taiwan

^3^Department of Biotechnology and Animal Science, National Ilan University, Ilan, Taiwan

^4^Department of Medical Imaging and Radiological Sciences, Chang Gung University, Taiwan

^5^Departments of Pathology, Tri-Service General Hospital, National Defense Medical Center, Taipei, Taiwan

^6^Graduate Institute of Aerospace and Undersea Medicine, National Defense Medical Center, Taipei, Taiwan

^7^Department of Medical Research, China Medical University Hospital, China Medical University, Taichung, Taiwan

*****Correspondence to: Prof. Kuo-Feng Hua. No. 1, Sec. 1, Shennong Road, Ilan 260, Taiwan. Fax: +886 3931-1526. E-mail address: kuofenghua@niu.edu.tw; or Prof. Shuk-Man Ka. No. 161, Sec. 6, Min-Quan E. Road, Taipei 114, Taiwan. Fax: +886 3935-4794. E-mail address: shukmanka@gmail.com.

**Full-length blots of Figure 2A**


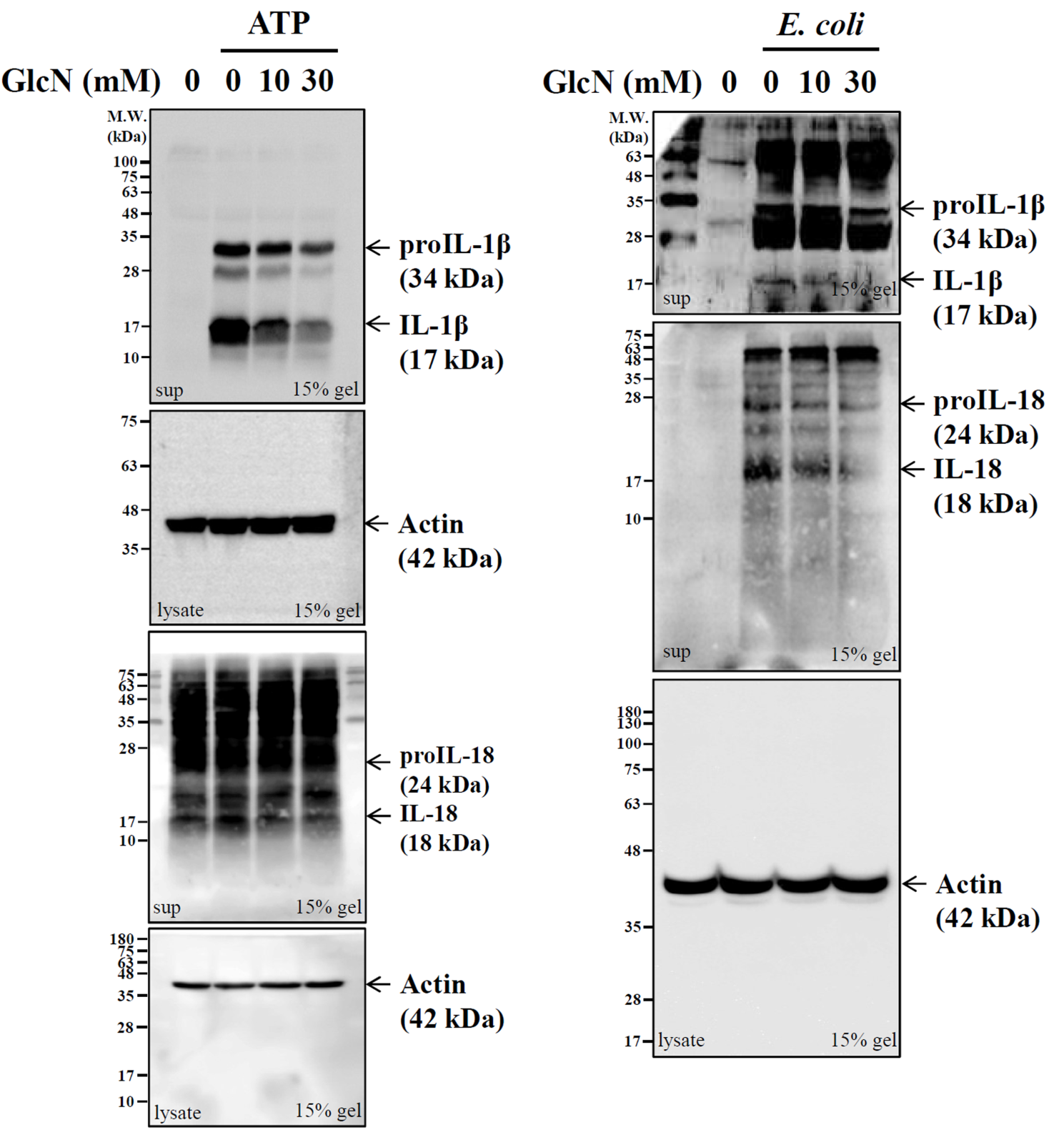


**Full-length blots of Figure 2B**

**
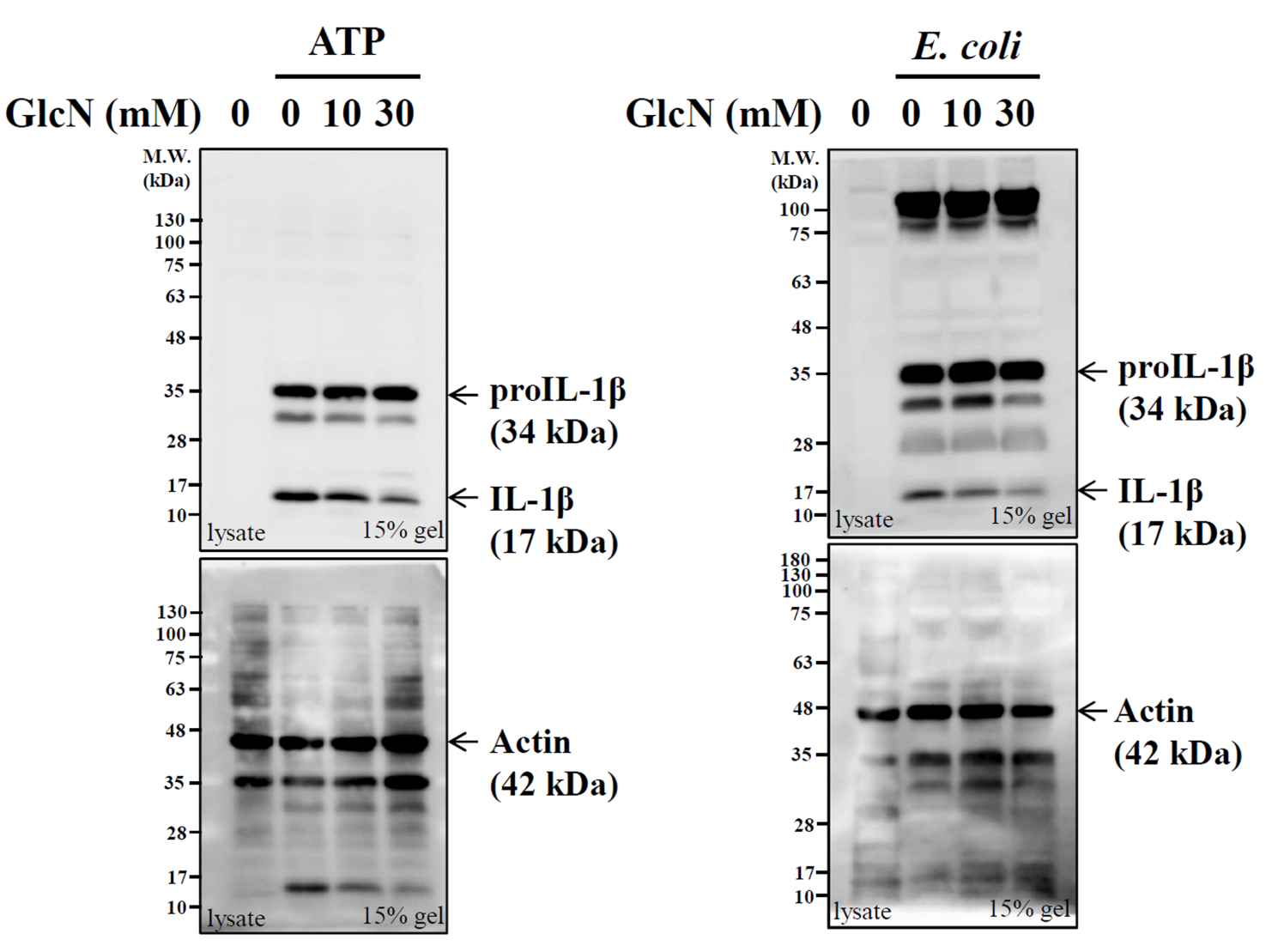
**

**Full-length blots of Figure 2C**

**
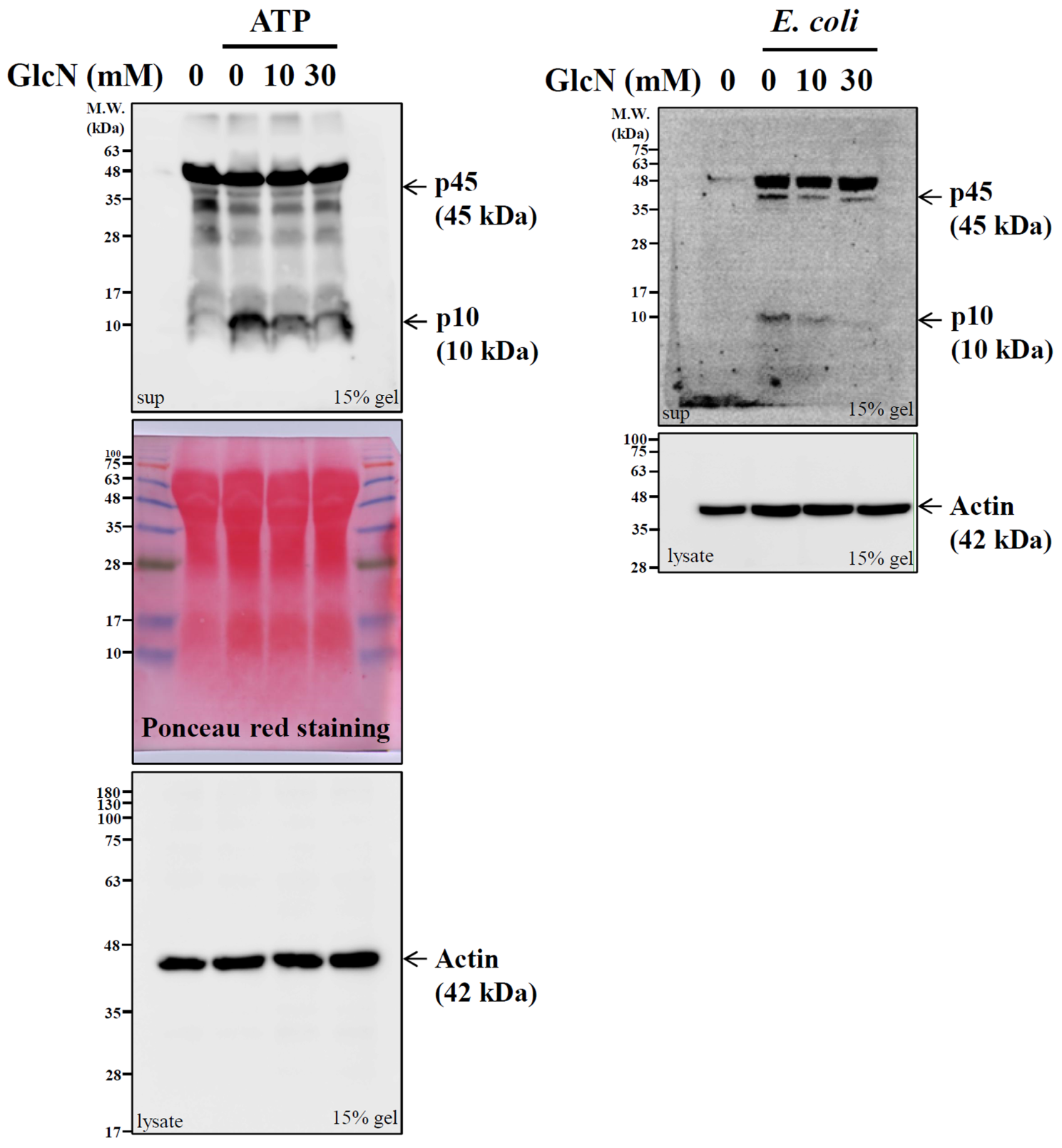
**

**Full-length blots of Figure 2D**


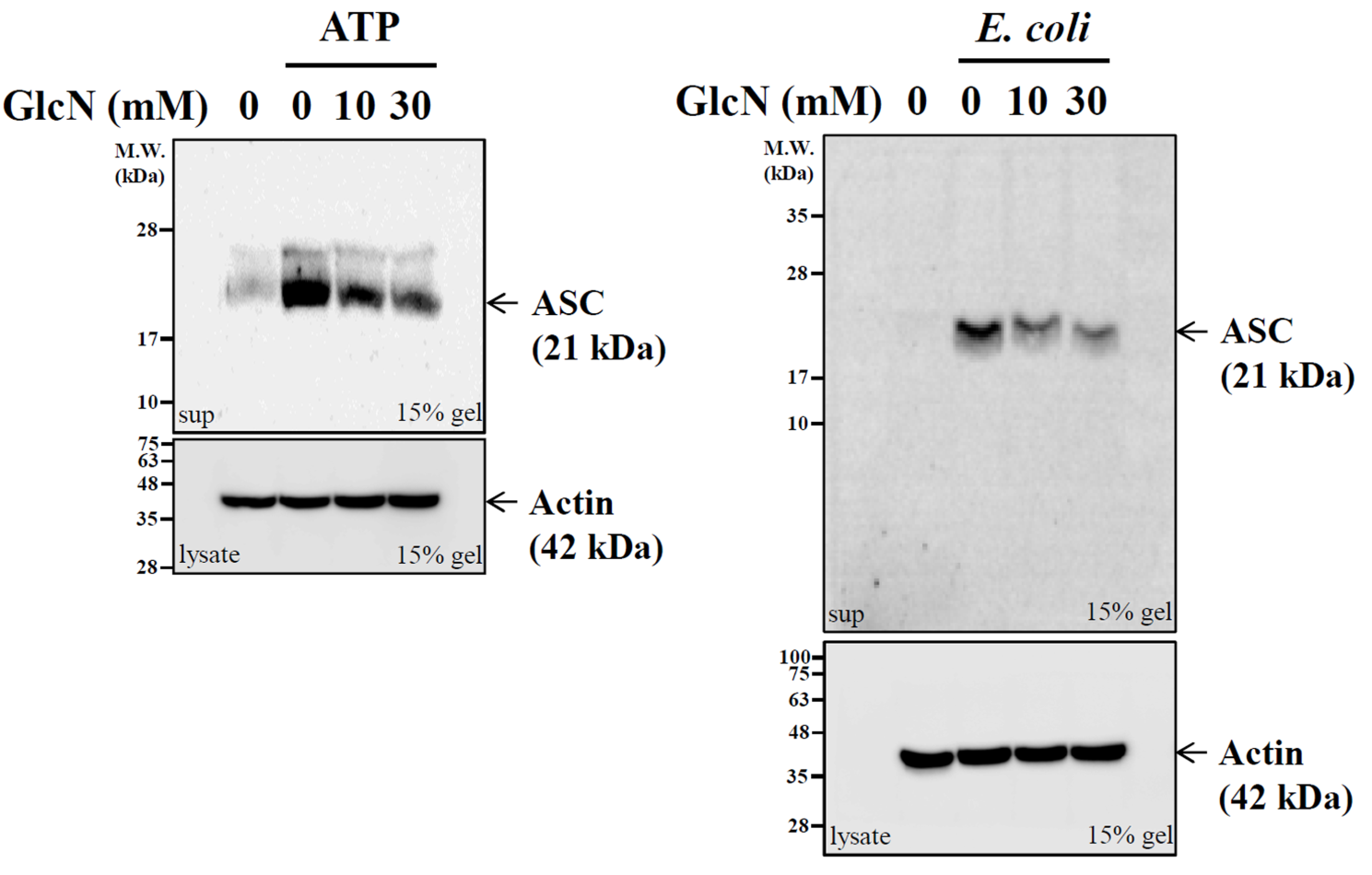


**Full-length blots of Figures 4A and 4B**

**
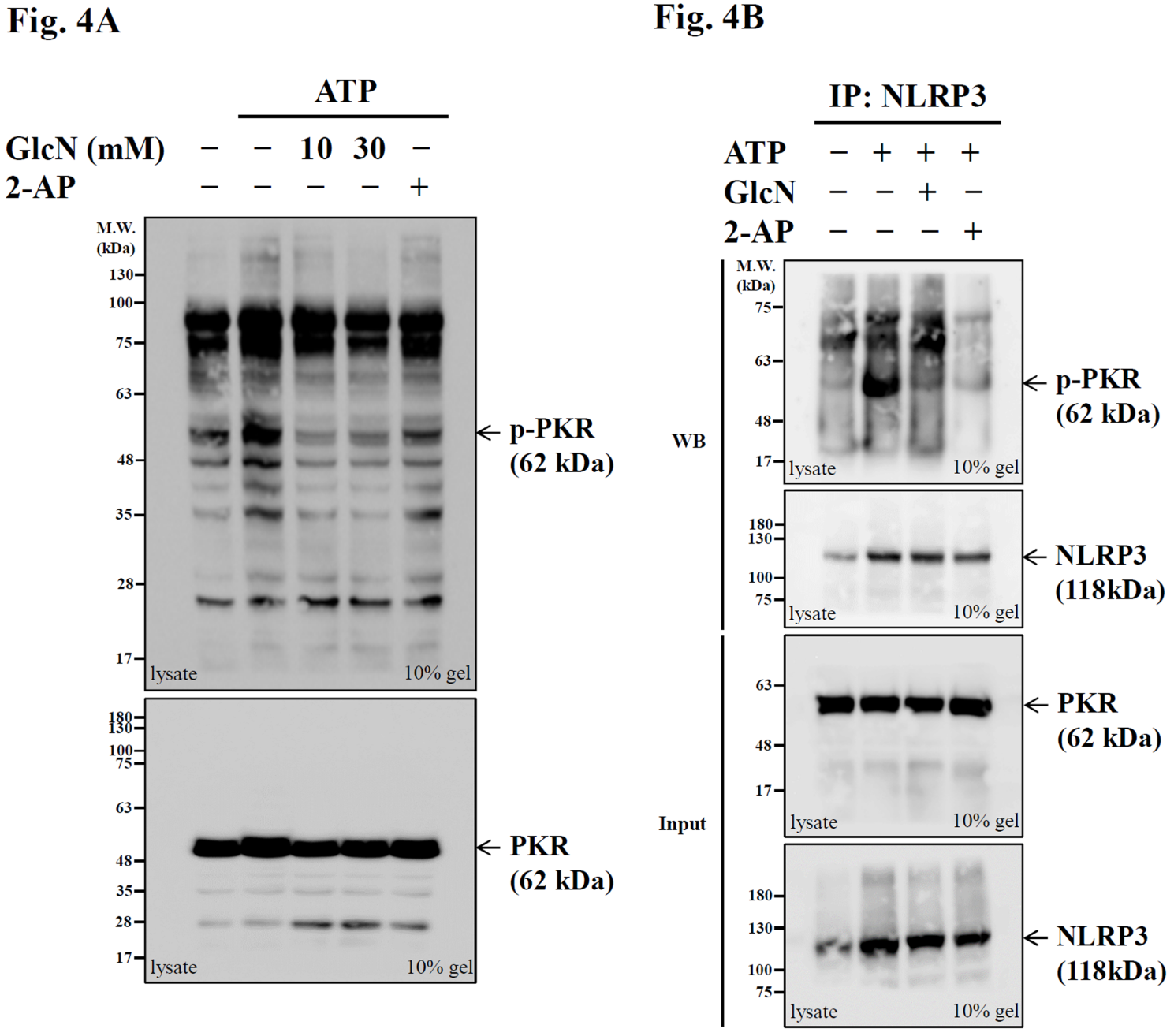
**

**Full-length blots of Figures 4C, 4D and 4E**

**
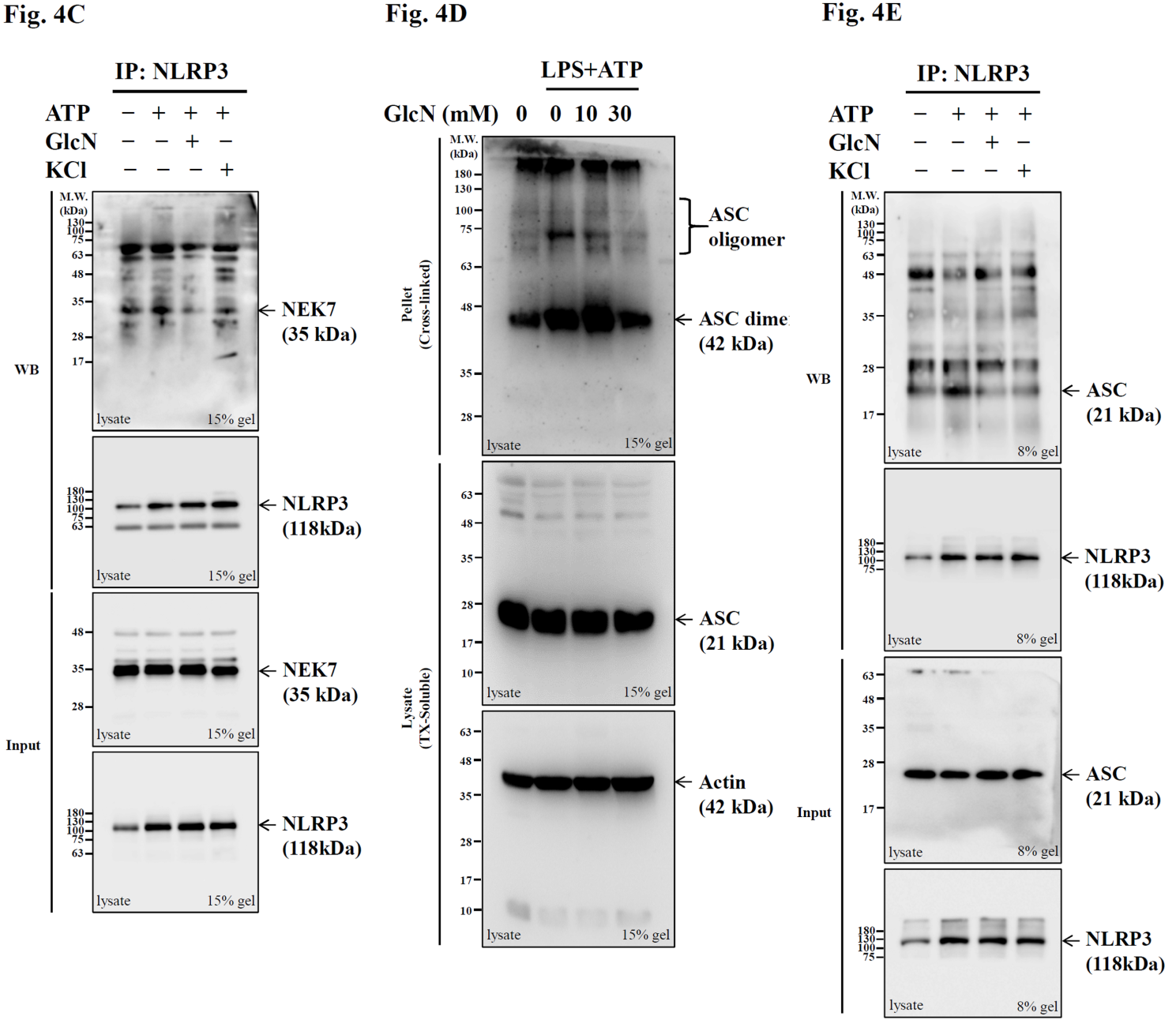
**

**Full-length blots of Figures 5A and 5B**

**
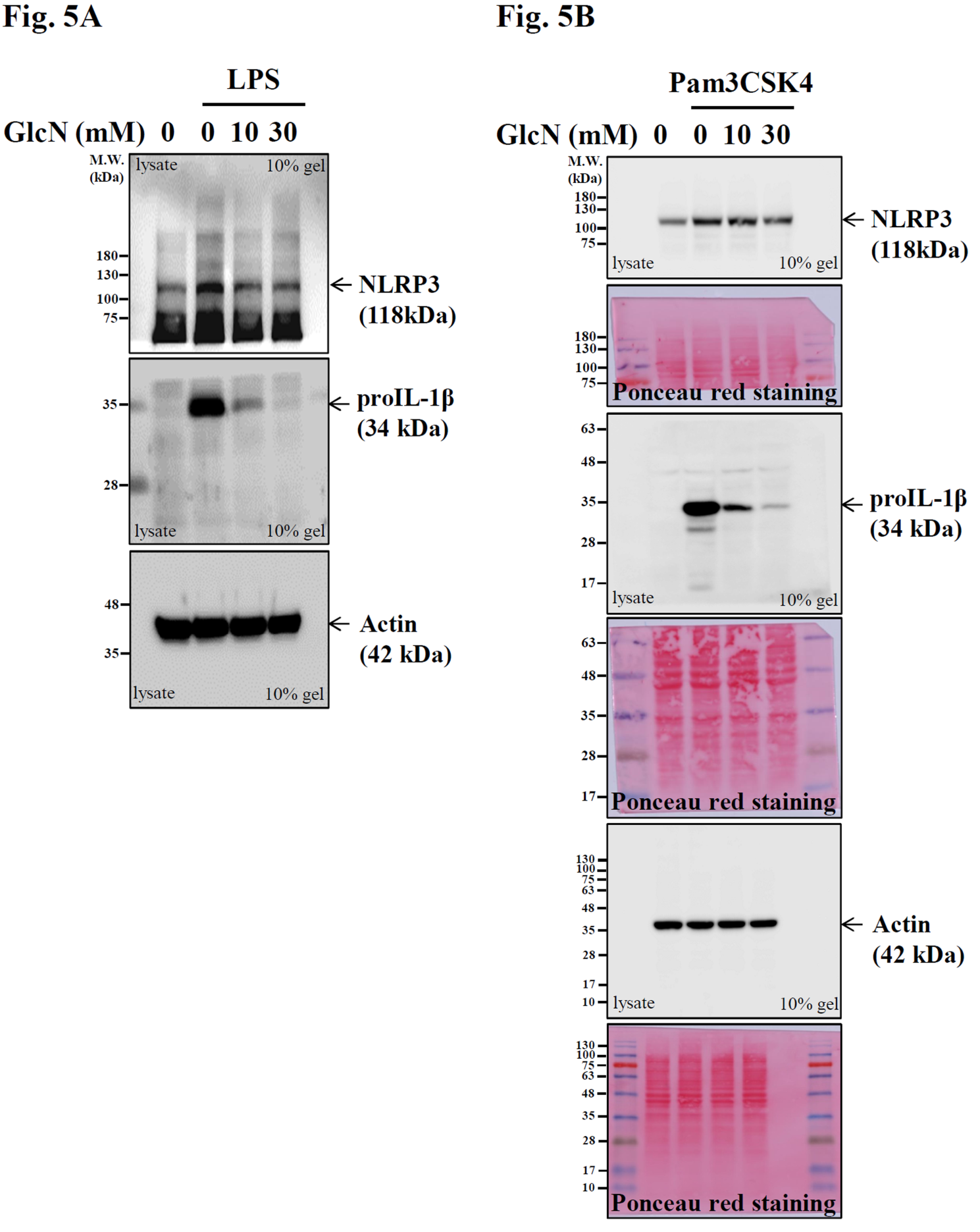
**

**Full-length blots of Figure 5E**


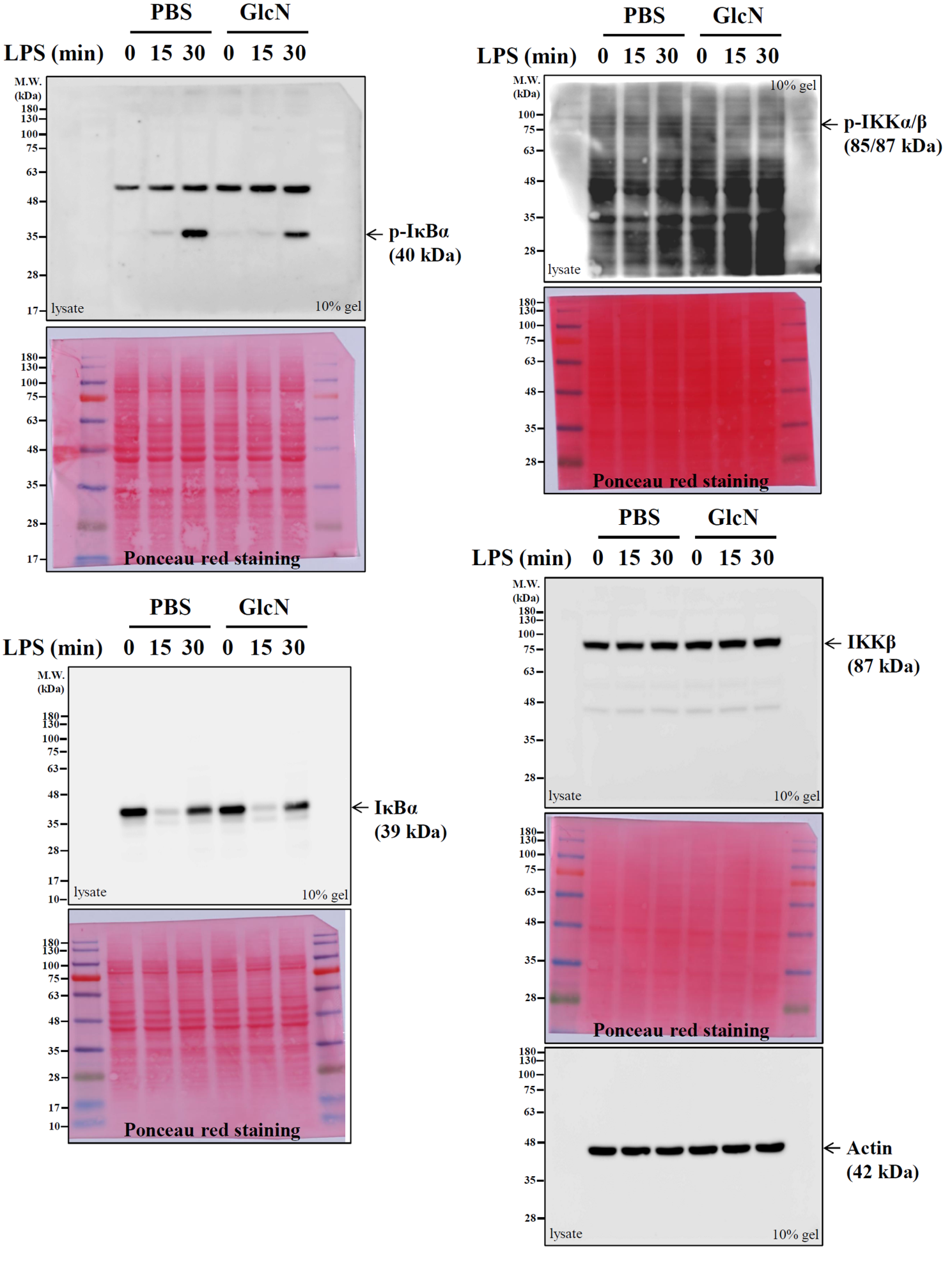


**Full-length blots of Figure 5F**


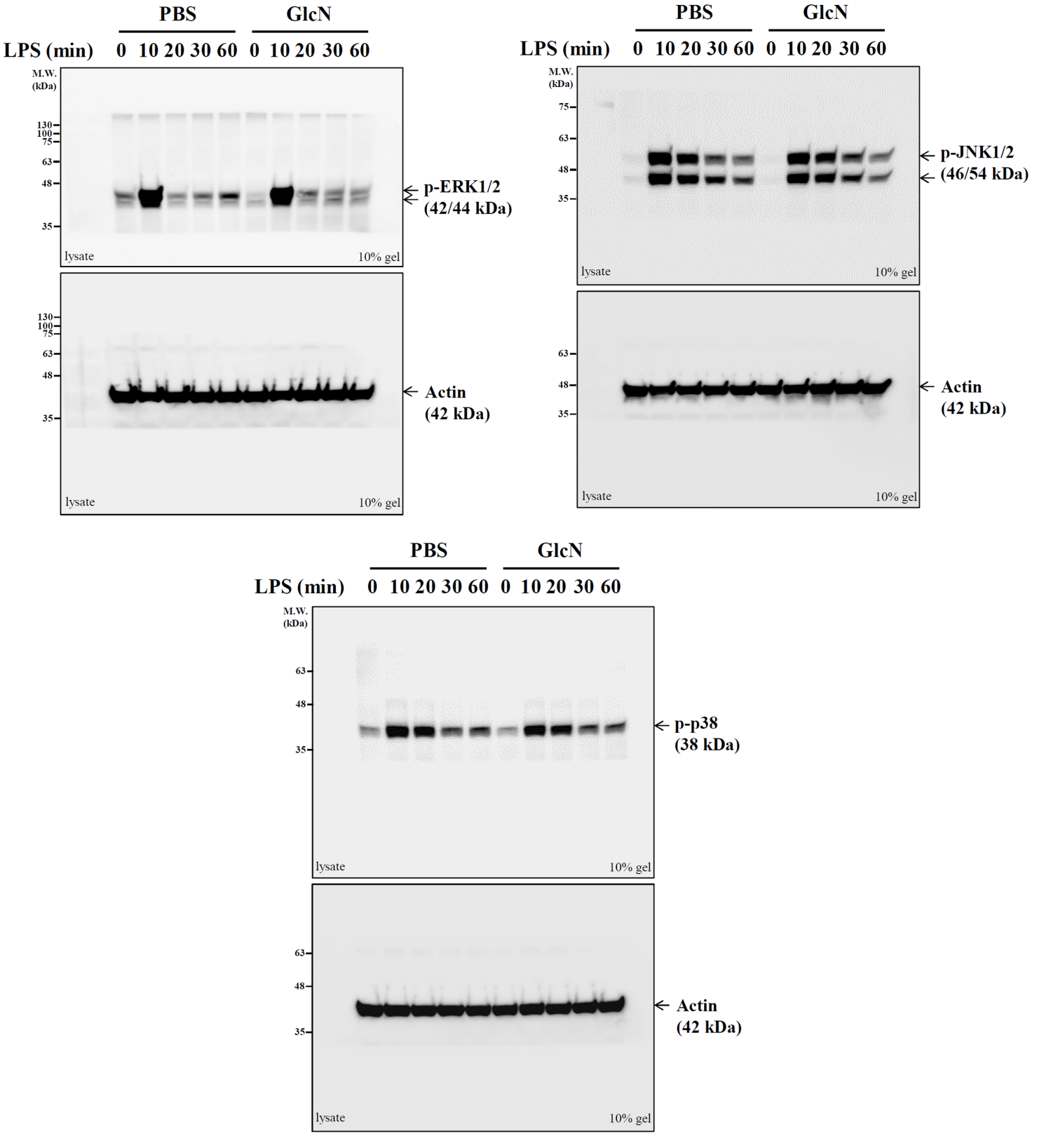

Supplement: Supplementary file 1 — Supplementary Info [file 41598_2019_42130_MOESM1_ESM.docx]
